# Dietary supplementation of *Citrus* bioflavonoids improves lactation performance in buffaloes during hot weather by regulating antioxidant capacity, immune function, and rumen microbes

**DOI:** 10.3389/fvets.2025.1707093

**Published:** 2025-11-03

**Authors:** Yinghui Li, Hanxing Yao, Chenglong Li, Mengwei Li, Fengming Chen, Xingguo Huang, Chengjian Yang

**Affiliations:** ^1^Guangxi Key Laboratory of Buffalo Genetics, Reproduction and Breeding, Guangxi Buffalo Research Institute, Nanning, Guangxi, China; ^2^College of Animal Science and Technology, Hunan Agricultural University, Changsha, Hunan, China; ^3^Yuelushan Laboratory, Changsha, Hunan, China; ^4^Hunan Provincial Key Laboratory of the TCM Agricultural Biogenomics, Changsha Medical University, Changsha, Hunan, China

**Keywords:** *Citrus* bioflavonoids, lactation performance, rumen microbes, immune function, dairy buffalo, heat stress

## Abstract

*Citrus* bioflavonoids (CB) are well recognized for their antioxidant, anti-inflammatory, and digestion-promoting properties. This study aimed to evaluate the effects of dietary CB supplementation on apparent nutrient digestibility, lactation performance, blood biochemical/immunological parameters, and the ruminal microbial community of dairy buffaloes during hot weather. A 35-day trial was conducted using 20 Mediterranean dairy buffaloes, which were randomly assigned to two groups: a control group (CON) fed a basal diet, and a CB-supplemented group (CB) fed the basal diet plus 20 g/d of CB. The results showed that compared with the CON group, CB supplementation significantly reduced (*p* < 0.05) the respiratory rate, and increased (*p***<** 0.05) the apparent digestibility of acid detergent fiber in buffaloes. Regarding lactation performance, CB supplementation significantly elevated (*p***<** 0.05**)** milk yield, 4% fat corrected milk, and the percentage of milk protein, lactose, and solids-not-fat; it also increased the concentrations of polyunsaturated fatty acids and conjugated linoleic acids in milk while decreasing (*p* < 0.05) the concentration of saturated fatty acids. For blood parameters, the CB group exhibited significantly higher (*p* < 0.05) blood catalase activity, immunoglobulin M concentration, and heat shock protein 70 concentration, as well as significantly lower (*p* < 0.05) concentrations of the pro-inflammatory cytokines tumor necrosis factor-*α*, interleukin (IL)-1β, and IL-6. In terms of ruminal function, CB supplementation significantly increased (*p* < 0.05) ruminal microbial crude protein concentration. There was a tendency toward higher (0.05 < *p* < 0.10) microbial *α*-diversity indices (Chao 1, Ace, and Shannon) in the CB group, accompanied by the enrichment of several bacterial genera within the family *Lachnospiraceae* and other saccharolyti*c* taxa, and the suppression of the genus *Segatella*. In conclusion, dietary supplementation with 20 g/d CB enhances the lactation performance of heat-stressed dairy buffaloes by improving the ruminal microenvironment and host health status.

## 1 Introduction

Global climate change has intensified the frequency and severity of heat waves, posing a significant threat to livestock production, particularly in tropical and subtropical regions where ambient temperatures often exceed the thermoneutral zone of ruminants. Heat stress disrupts the physiological homeostasis of animals, triggering a cascade of adverse responses including elevated core body temperature, increased respiratory and heart rates, and suppressed feed intake (1, 2). Among ruminants, buffaloes (*Bubalus bubalis*) are inherently more susceptible to heat stress due to their unique anatomical and physiological characteristics: they possess a lower density of sweat glands, a thicker epidermis, and a darker coat, all of which hinder efficient heat dissipation through evaporation and radiation (3, 4). Under prolonged heat stress, buffaloes experience heightened oxidative stress, as the overproduction of reactive oxygen species (ROS) overwhelms the endogenous antioxidant defense system, damaging cellular lipids, proteins, and DNA (5). Concurrently, heat stress suppresses immune function by altering cytokine profiles and reducing lymphocyte activity, thereby increasing susceptibility to infectious diseases (6). These physiological impairments, coupled with reduced nutrient utilization due to altered rumen fermentation, collectively contribute to poor lactation performance, making heat stress a critical constraint to sustainable buffalo dairy production in hot climates.

*Citrus aurantium L*., commonly known as trifoliate orange in traditional Chinese medicine, is a deciduous shrub whose dried immature fruit (*Fructus aurantii*) has been widely used for centuries to treat digestive and metabolic disorders (7). Modern phytochemical studies have identified *Citrus* bioflavonoids (CB) as the primary bioactive components of *Fructus Aurantii*, including hesperidin, naringin, neohesperidin, and quercetin, which are renowned for their antioxidant, anti-inflammatory, and immunomodulatory properties (7). Studies focusing on citrus-derived flavonoids have demonstrated their protective role in rumen and overall health of ruminants. Paniagua et al. (8, 9) reported that *Citrus* flavonoids supplementation can enhance feed efficiency, alter rumen macroscopic characteristics, and improve rumen wall health in Holstein bulls. Similarly, other studies have shown that C*itrus* flavonoid extracts reduce bacterial endotoxin and systemic inflammation in dairy cows, thereby improving immune-metabolic status and modulating the hindgut microbiome (10, 11). These findings highlight the potential of CB as natural alternatives to synthetic anti-stress agents, aligning with the growing demand for sustainable and antibiotic-free livestock production. However, research specifically investigating the effects and underlying mechanisms of CB on heat-stressed buffaloes remains scarce.

We hypothesized that dietary supplementation with CB would improve the fermentation profile, antioxidant status, and immune function of dairy buffaloes under hot weather conditions by regulating rumen microbes, resulting in a positive impact on their lactation performance. Therefore, the current study aimed to evaluate the effects of dietary CB supplementation on lactation performance, blood antioxidant and immune indices, rumen fermentation profiles, and rumen microbial communities.

## 2 Materials and methods

### 2.1 Animal ethics statement

All animal experimental procedures were approved by the Institutional Animal Care and Use Committee of the Guangxi Buffalo Research Institute, Chinese Academy of Agricultural Sciences (Nanning, Guangxi, China), with the approval protocol No. 20240801L.

### 2.2 *Citrus* bioflavonoids

The *Citrus* extract used in this study was kindly provided by Lianyuan Kanglu Biotechnology Co., Ltd. (Loudi, Hunan, China), with a total flavonoid content of 22.28%. CB were extracted from dried bitter orange (*Citrus aurantium* L.) following the optimized protocol described below. In brief, dried bitter orange was first crushed and sieved through a 40-mesh sieve to obtain a uniform *Citrus* powder firstly. 1 kg of the *Citrus* powder was mixed with 10 L of 70% (v/v) ethanol, and ultrasonic extraction was performed for 35 ± 5 min. Then, the mixture after ultrasonic treatment was subjected to reflux extraction to maximize the recovery of flavonoids. The resulting extract was concentrated and lyophilized for 24 h to obtain the crude CB extract. The total flavonoid content of the crude CB extract (22.28%) was determined by HPLC, including naringin (7.38%), hesperidin (5.62%), neohesperidin (3.05%), and nobiletin (1.26%).

### 2.3 Animals, diets and experimental design

This study was conducted from August to September 2024 at the Guangxi Buffalo Research Institute, Nanning, South China (22°53′22.59″N, 108°21′51.19′′E). A total of 20 Mediterranean dairy buffaloes with similar body weight (600 ± 25 kg), parity (2.69 ± 1.30), and lactation stage (120 ± 20 d) were enrolled in this study under a completely randomized design. Buffaloes were randomly assigned to two dietary treatments: basal diet (CON group) or basal diet supplemented with 20 g/d of CB per buffalo (CB group). The dose of CB was determined based on a prior *in vitro* rumen simulation technique experiment conducted by our team. In that *in vitro* study, supplementing CB at 0.15% of dietary dry matter (DM) in total mixed ration (TMR) yielded the optimal rumen fluid fermentation performance for dairy buffaloes (unpublished data). For the current *in vivo* study, the CB dose was adjusted to 0.1% of the average dry matter intake (DMI, approximately 14 kg/head·d) of dairy buffaloes, resulting in a final dose of 20 g/head·d.

The CB product was top-dressed daily and mixed with a small amount of total mixed ration (TMR) during morning feeding (before milking), and each buffalo was monitored for feed leftover. The experiment lasted for 5 weeks, including a 1-week adaptation period. All buffaloes were fed the basal TMR twice daily (05:00 and 15:00) for *ad libitum* intake. The TMR was formulated to meet the nutritional requirements of dairy buffaloes, consisting of *Pennisetum purpureum* (elephant grass), corn silage, brewer’s grains, and a concentrated feed mixture. Details of the chemical composition of the experimental diet are given in Table 1. Individual DMI was calculated daily by weighing the offered feed and orts for each buffalo. Fresh water was available *ad libitum* via individual water troughs.

**Table 1:** Composition and nutritional level in the basal diet (DM basis).

Items	Content
Ingredients, %	
*Pennisetum purpureum* Schumach.	12.00
Corn silage	29.28
Brewer’s grains	40.51
Concentrated feed mixture^1^	17.21
Premix^2^	0.50
NaCl	0.50
Total	100.00
Nutrient levels^3^, %	
GE, MJ/kg	17.10
CP	18.94
Crude ash	7.18
NDF	45.16
ADF	23.67
Ca	1.83
P	0.37

1 The concentrated feed mixture was purchased from a livestock and poultry company in Nanning, with the following nutritional composition (as-fed basis): DM 91.50%, CP 20.05%, crude ash 7.93%, NDF 15.44%, ADF 6.52%, and GE 19.04 MJ/kg.

2 Per kg premix contained the following: VA 550,000 IU, VE 3,000 IU, VD 3150,000 IU, Fe (as ferrous sulfate) 4.0 g, Cu (as copper sulfate) 1.3 g, Mn (as manganese sulfate) 3.0 g, Zn (as zinc sulfate) 6.0 g, Co (as Cobalt sulfate) 80.0 mg.

3 All the nutrient levels are measured values.

Aside from time for exercise and swimming, buffaloes were housed individually in an open-sided shed. To exercise, the buffaloes were set free in an adjacent open yard with a stocking density of 15 m^2^/buffalo. Fans were installed in the buffalo barn to improve airflow. Buffaloes were allowed 30 min of swimming time before milking. All buffaloes were machine-milked twice daily (05:00 and 14:00), and individual milk yield was recorded manually immediately after milking.

### 2.4 Recording of meteorological data and physiological parameters of buffalo

An online dust monitoring system (Shenzhen Greenforze Environmental Technology Co., Ltd., Shenzhen, China) was used to record daily meteorological data in real-time, mainly including air temperature (AT, °C) and relative humidity (RH, %), at an interval of 30 min. The device was installed at a height equal to the height of the animal’s back and can measure a temperature range from −20 to 80 °C with a precision of ±0.5 °C (error rate ±1%).

Recording of physiological parameters was performed in the morning and afternoon twice a week (on Monday and Thursdays). The rectal temperature (RT, °C), body surface temperature (BST, °C), and respiratory rate (RR, breaths/min) of buffaloes were recorded in the shed in the morning (8:00–9:00 a.m.) and afternoon (14:30–15:30 p.m.). The rectal temperature was recorded using an animal rectal thermometer (GLA 700, GLA Corporation, San Luis Obispo, CA 93401, U.S.), and the stabilized maximum temperature was taken as the rectal temperature value by keeping the thermometer in the rectum for 2 min. Body surface temperature was detected using an animal infrared thermometer (HRQ-S60, Zhengzhou Haorunqi Electronic Technology Co., Ltd., Zhengzhou, Henan, China), and the average temperature value of three body sites (forehead, left chest, and left abdomen) was taken as the body surface temperature of the buffalo. Respiratory rate (breathes/min) was recorded by manual counting using a stopwatch, observation chest and abdomen movements for 2 min, and the average value per minute was calculated as the RR.

### 2.5 Evaluation of thermal comfort state in buffalo

The thermal comfort state of buffaloes was evaluated using the paired index models (E3 for environment conditions and P3 for physiological status) proposed by Li et al. (12), which have been validated as practical and accurate for assessing the thermal comfort of dairy buffaloes under hot and humid climate. The calculation formulas for the two indices are as follows: E3 (Environmental index model) = 1.016 × AT (°C) + 0.139 × RH (%); P3 (physiological index model) = 0.654 × BST (°C) + 0.381 × RR (breaths/min). Thermal comfort levels of buffaloes are categorized into four grades based on the E3 and P3 values, with thresholds and corresponding states defined as follows: comfort (E3 ≤ 37.15, P3 ≤ 25.30); danger (37.15 < E3 ≤ 44.06, 25.30 < P3 ≤ 28.64); stress (44.06 < E3 < 50.97, 28.64 < P3 < 31.98); emergency (E3 ≥ 50.97, P3 ≥ 31.98).

### 2.6 Apparent total tract digestibility (ATTD) analysis

Feed samples were collected weekly throughout the experimental period and immediately stored at −20 °C. Samples were composited by period, dried at 65 °C for 48 h, ground using a multifunctional pulverizer (Old Bank Boou Hardware Factory, Zhejiang, China), and sieved through a 40-mesh stainless steel sieve (manufactured by Tianxing Wusi Yarn Sieve Factory, Shangyu City, Zhejiang, China). The ground sample was stored at −20 °C until subsequent nutrient analysis. The dry matter (DM), crude protein (CP), ether extract (EE), acid detergent fiber (ADF), neutral detergent fiber (NDF), and acid-insoluble ash of feed were determined according to the Association of Official Analytical Chemists (AOAC, 1995).

Fecal samples were collected during the last 3 consecutive days of the experimental period, with two collections per day (07:00 and 17:00). Feces were weighed and mixed daily, and 10 mL of 10% H_2_SO_4_ was added to each daily mixed fecal sample (per 100 g of fresh feces) to fix nitrogen, then stored at −20 °C for further analysis. Subsequently, thawed fecal samples were dried at 65 °C for 48 h and ground to pass through a 40-mesh sieve. Concentrations of CP, EE, ADF, and NDF in feces were analyzed as described above.

### 2.7 Milk yield and composition

Morning (05:00) and afternoon (14:00) milk yield was recorded daily for each buffalo, and milk samples for determination of milk composition were collected weekly. Fresh milk samples were used to analyze milk composition, including milk protein, fat, lactose, milk urea nitrogen (MUN), total solids (TS), solids-not-fat (SNF), free fatty acids (FFA) for morning and afternoon separately using MilkoScan™ F120 (FOSS, Hillerød, Denmark). Subsequently, 4% fat-corrected milk (4% FCM) was calculated according to NRC (2001) using the following equation: 4% FCM = 0.4 × milk yield (kg/d) + 0.15 × milk fat percentage (%) × milk yield (kg/d).

Milk samples collected twice a day were mixed proportionally to morning and afternoon milk yield for each week and stored at −20 °C. The pooled samples were quickly sent to the Shanghai Sensichip Biotech Co., Ltd. for analysis of medium- and long-chain fatty acid profile using chromatography-mass spectrometry (GC–MS), which was performed with a Thermo Scientific Trace 1,300 gas chromatograph connected to a Thermo Scientific ISQ7000 single quadrupole mass spectrometer selective detector (Trace1300- ISQ7000, Thermo Fisher Scientific, Waltham, MA, USA). A DB-5MS chromatographic column was used (60 m × 0.25 mm inner diameter and 0.25 μm film thickness; J&W Scientific, USA).

### 2.8 Blood samples collection and analysis

Before morning feeding on the last day of the experimental period, blood samples (approximately 10 mL) were collected from the coccygeal vein of buffaloes using disposable vacuum blood collection tubes (non-anticoagulant tubes) (Shandong Aosaite Medical Devices Co., Ltd.). The blood samples were then centrifuged at 4 °C (1,500 × g) for 15 min, and serum was harvested and stored at −80 °C until further analysis. Simultaneously, 10 mL blood samples were collected using heparin sodium-anticoagulated tubes and centrifuged at 4 °C (3,500 × g) for 15 min. The resulting plasma was then stored at −80 °C until further analysis.

Serum concentrations of total protein (TP), albumin (ALB), alanine transaminase (ALT), aspartate aminotransferase (AST), alkaline phosphatase (ALP), creatine kinase (CK), lactate dehydrogenase (LDH), urea nitrogen (UN), glucose (GLU), total cholesterol (TC), triglyceride (TG), immunoglobulin G (IgG), IgM, IgA, insulin (INS), triiodothyronine (T3), and tetraiodothyronine (T4) were determined using an automatic blood biochemical analyzer (Cobas c311, Roche, Switzerland), according to the manufacturer’s instructions (Roche Biochemical Kit). Plasma superoxide dismutase (SOD) and catalase (CAT) activities, as well as total antioxidant capacity (T-AOC) and malondialdehyde (MDA) levels, were measured using commercial kits (Nanjing Jiancheng Bioengineering Institute) in strict compliance with the operating procedures. The concentrations of serum amyloid A (SAA), heat shock protein 70 (HSP70), tumor necrosis factor-*α* (TNF-α), interferon-*γ* (IFN-γ), interleukin-1beta (IL-1β), interleukin-2 (IL-2), and interleukin-6 (IL-6) were detected using ELISA kits provided by Jiangsu Jingmei Biotechnology according to the manufacturers’ guidelines.

### 2.9 Collection of ruminal contents and processing

On the last day of the experiment before morning feeding, ruminal contents samples (500 mL) were collected in sterilized plastic bottles from buffaloes using a stainless-steel stomach tube. After collection, samples were immediately transferred to the laboratory for further analysis. Subsequently, the ruminal contents were strained through two layers of cheesecloth and subsamples of ruminal contents for determination of volatile fatty acids (VFA), ammonia nitrogen (NH_3_-N) and microbial crude protein (MCP) were stored at −20 °C. Subsamples for DNA extraction were stored at −80 °C until further processing.

### 2.10 Determination of rumen fermentation parameters

After the collection of ruminal contents, pH was measured immediately using a pH meter (HI 9024C; HANNA Instruments, Woonsocket, RI, USA). A subsample of rumen fluid (4 mL) was acidified with 4 mL of 0.2 mol/L HCl and stored at −20 °C for determination of NH_3_-N using the indophenols method. MCP content was analyzed with a spectrophotometer at 595 nm using 1 mg/mL bovine serum albumin solution (Sigma-Aldrich Co., LLC, St. Louis, MO, USA) as the standard. The concentrations of VFA, including acetate, propionate, isobutyrate, butyrate, isovalerate, and valerate, were measured using a gas chromatography (GC) system (Agilent 7890A, Agilent Technologies, USA).

### 2.11 DNA extraction from ruminal contents and sequencing of the 16S rRNA gene

Total genomic DNA from rumen fluid was extracted using an E.Z.N.A Soil DNA Kit (Omega Bio-Tek, Norcross, GA, USA). DNA concentration and purity were determined using a NanoDrop 2000 spectrophotometer (Thermo Scientific, Wilmington, DE, USA). High throughput (Illumina MiSeq) sequencing of the 16S rRNA gene was carried out using barcoded primers targeting the V3–V4 region. Amplicons were extracted from 2% agarose gels and purified using the QIAquick PCR Purification Kit (Qiagen, Hilden, Germany), and library construction was performed according to Illumina’s instructions.

Paired-end reads were assigned to the samples based on their unique barcodes and truncated by removing the barcodes and primer sequences. The raw read pairs were overlapped and merged using FLASH (v1.2.11), then imported into QIIME 2 for demultiplexing. Quality control, denoising, removal of chimeric sequences, and generation of amplicon sequencing variants (ASVs) were performed using the QIIME2 DADA2 plugin. The SILVA database (version 138) was used for ASV classification and annotation analysis, and the microbial species composition at different taxonomic levels (phylum, class, order, family, genus, and species) was obtained. Several *α*-diversity indices, including Chao 1, Ace, Shannon, and Simpson indices, were calculated using QIIME 2. Microbial *β*-diversity was determined using distance matrices generated from Bray-Curtis analysis, principal coordinate analysis (PCoA), and analysis of similarities (ANOSIM). Linear discriminant analysis effect size (LEfSe) was used to identify significantly different taxa between the two treatment groups.

### 2.12 Statistical analysis

Data were analyzed using independent samples t-test with SPSS version 23 (IBM Corp., Armonk, NY, USA). Differences in microbial composition and *α*-diversity between the two groups were compared using Welch’s *t*-test, and *p* values were corrected by false-discovery rate adjustment. All results are reported as means ± standard error of the mean (SEM), with significant differences declared at *p* < 0.05 and a trend of difference defined at 0.05 ≤ *p* < 0.10.

## 3 Results

### 3.1 Thermal comfort state in dairy buffalo during the experiment

**Figure 1:**
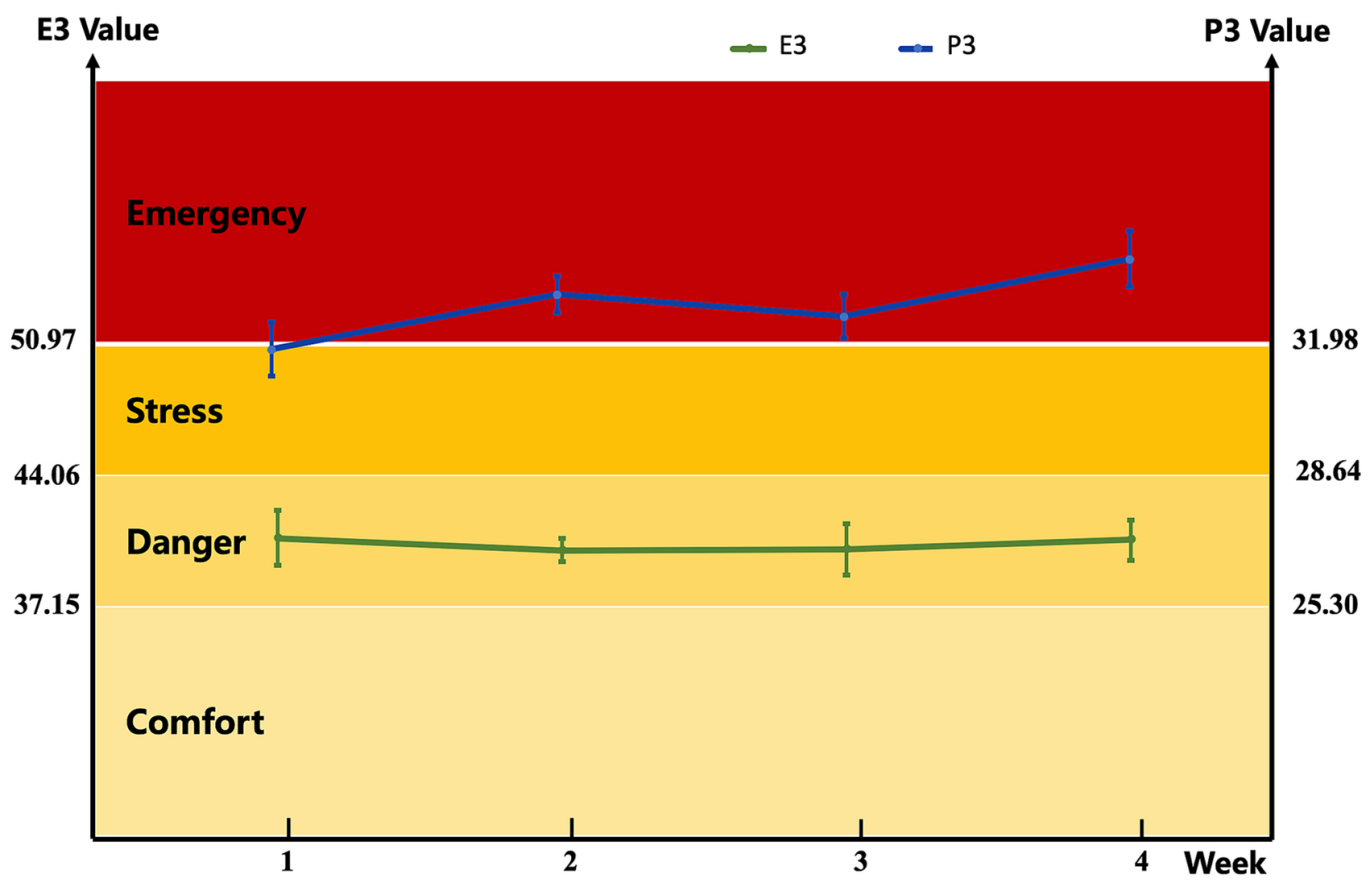
Environmental index and physiological index models of dairy buffaloes during the experiment. The environment variables and the buffalo physiological index variables can be derived from this index model (practical model) as follows: E3 (Environmental index model) = 1.016 × AT (°C) + 0.139 × RH (%); P3 (physiological index model) = 0.654 × BST (°C) + 0.381 × RR (breaths/min). Buffalo status is comfort (E3 ≤ 37.15; P3 ≤ 25.30), danger (37.15 < E3 ≤ 44.06; 25.30 < P3 ≤ 28.64), stress (44.06 < E3 < 50.97; 28.64 < P3 < 31.98), emergency (E3 ≥ 50.97; P3 ≥ 31.98). AT, air temperature; RH, relative humidity; BST, body surface temperature; RR, respiratory rate.

During the experimental period, the E3 ranged from 40.71 to 41.30, indicating that the thermal comfort state of the buffaloes was in the “danger” grade; meanwhile, the P3 varied from 31.30 to 38.00, with most values exceeding the “emergency” threshold (P3 ≥ 31.98), indicating that the dairy buffaloes were primarily in an emergency state (Figure 1). Notably, compared with E3 (which integrates AT and RH), P3 (combining BST and RR) more intuitively reflected the actual heat stress status of the buffaloes, as it directly mirrors the host’s physiological responses to thermal stress. Collectively, the E3 and P3 data confirmed that the dairy buffaloes in this experiment experienced relatively severe and persistent heat stress throughout the trial.

### 3.2 Physiological indices

As shown in Table 2, the RR of buffaloes in the CB group was significantly lower (*p* < 0.05) than that in the CON group, with a reduction of 17.0%. Meanwhile, compared to the CON group, CB supplementation tended to decrease the BST of the dairy buffaloes (*p* = 0.07). In contrast, dietary CB supplementation had no effect on the RT of the dairy buffaloes (*p* > 0.05).

**Table 2:** Effects of dietary with *Citrus* bioflavonoids (CB) supplementation on physiological indices of dairy buffaloes during hot weather.

Items	Treatment groups^1^		*p*-value
	CON	CB	
BST, °C	35.93 ± 0.04	35.77 ± 0.07	0.070
RT, °C	38.62 ± 0.06	38.74 ± 0.07	0.221
RR, breaths/min	32.10 ± 1.09^a^	27.57 ± 1.30^b^	0.015

BST, body surface temperature; RR, respiratory rate; RT, rectal temperature.

^a,b^Mean values within a row with different superscripts differed (*p* < 0.05).

1 CON, control diet with no CB supplementation; CB, control diet with 20 g/d CB supplementation for per buffalo.

### 3.3 Feed intake and nutrient apparent digestibility

Results revealed no significant effect (*p* > 0.05) of CB supplementation on the DMI of the dairy buffaloes (Table 3). Similarly, no differences were observed between the CON and CB groups in the apparent digestibility of nutrients including CP, EE, and NDF (*p* > 0.05). Nevertheless, the apparent digestibility of ADF in the CB group was 6.89% higher (*p* < 0.05) than that in the CON group (Table 3).

**Table 3:** Effects of dietary with *Citrus* bioflavonoids (CB) supplementation on the dry matter intake and nutrient apparent digestibility of dairy buffaloes during hot weather.

Items	Treatment groups^1^		*p*-value
	CON	CB	
DMI, kg/d	15.03 ± 0.30	15.16 ± 0.80	0.882
CP, %	58.56 ± 1.84	59.53 ± 1.500	0.685
EE, %	60.98 ± 2.48	62.57 ± 2.52	0.658
ADF, %	33.66 ± 1.35^b^	40.55 ± 1.97^a^	0.010
NDF, %	58.11 ± 1.05	57.18 ± 1.80	0.663

DMI, dry matter intake; CP, crude protein; EE, ether extract; ADF, acid detergent fiber; NDF, neutral detergent fiber.

^a,b^Mean values within a row with different superscripts differed (*p* < 0.05).

1 CON, control diet with no CB supplementation; CB, control diet with 20 g/d CB supplementation for per buffalo.

### 3.4 Milk production and composition

The effects of CB supplementation on milk production and composition of dairy buffaloes during hot weather are summarized in Table 4. Compared to the CON group, CB supplementation significantly increased (*p* < 0.05) milk yield, 4% FCM, and several milk composition parameters including milk protein, milk lactose, SNF, and FFA with. However, no differences in milk fat, MUN, or TS contents were observed between the CON and CB groups (*p* > 0.05).

**Table 4:** Effects of dietary with *Citrus* bioflavonoids (CB) supplementation on milk production and composition in dairy buffaloes during hot weather.

Items	Treatment groups^1^		*p*-value
	CON	CB	
Milk yield, kg/d	6.76 ± 0.03^b^	7.32 ± 0.04^a^	< 0.01
4%FCM^2^, kg/d	10.66 ± 0.05^b^	11.74 ± 0.07^a^	< 0.01
Milk protein, %	4.59 ± 0.02^b^	4.77 ± 0.02^a^	< 0.01
Milk fat, %	7.85 ± 0.46	8.03 ± 0.37	0.772
Milk lactose, %	5.01 ± 0.02^b^	5.17 ± 0.02^a^	0.017
MUN, mg/dL	21.80 ± 0.88	20.55 ± 0.81	0.312
TS, %	18.34 ± 0.50	18.51 ± 0.40	0.800
SNF, %	10.26 ± 0.03^b^	10.44 ± 0.02^a^	< 0.01
FFA, mmol/L	0.41 ± 0.01^b^	0.49 ± 0.01^a^	< 0.01

MUN, milk urea nitrogen; TS, total solids; SNF, milk solids-not-fat; FFA, Free fatty acids; FCM, fat corrected milk.

^a,b^Mean values within a row with different superscripts differed (*p* < 0.05).

1 CON, control diet with no CB supplementation; CB, control diet with 20 g/d CB supplementation for per buffalo.

2 4%FCM = 0.4 × milk yield (kg/d) + 0.15 × milk fat (%) × milk yield (kg/d).

### 3.5 Milk fatty acid composition

In the present study, we quantified 38 major fatty acids including 15 saturated fatty acids (SFA), 10 monounsaturated fatty acids (MUFA), and 13 polyunsaturated fatty acids (PUFA) via GC–MS analysis. Our study revealed that the major SFA in buffalo milk were C12:0, C21:0, and C10:0, while the major unsaturated fatty acid (UFA) were C18:2 (10-TRANS, 12-CIS), C18:1n9t, and C20:5n3 (eicosapentaenoic acid, EPA) (Table 5). Compared to the CON group, the CB group exhibited a significant decrease in the contents of major SFA (C14:0, C15:0, C16:0, and C18:0) and a significant increase in the contents of MUFA (C20:1 and C22:1n9) and PUFA (C18:2(10-TRANS, 12-CIS), C20:3n3, and C22:5n3) in milk (*p* < 0.05). Consequently, dairy buffaloes receiving CB had lower total SFA contents and SFA/UFA ratio in milk, as well as higher total UFA, PUFA, and CLA contents, than those in the CON group (*p* < 0.05).

**Table 5:** Effects of dietary with *Citrus* bioflavonoids (CB) supplementation on milk fatty acids composition in dairy buffaloes during hot weather.

Items	Treatment groups^1^		*p*-value
	CON	CB	
C8:0, μg/mL	13.65 ± 2.04	17.36 ± 1.70	0.143
C10:0, μg/mL	313.32 ± 28.25	338.17 ± 27.25	0.535
C11:0, μg/mL	8.62 ± 1.75	9.37 ± 1.18	0.728
C12:0, μg/mL	602.12 ± 44.10	587.57 ± 39.83	0.809
C13:0, μg/mL	56.08 ± 7.99	60.81 ± 6.30	0.648
C14:1n5, μg/mL	24.46 ± 3.67	29.88 ± 3.38	0.291
C14:0, μg/mL	304.46 ± 25.82^a^	87.79 ± 9.39^b^	< 0.01
C15:1n5, μg/mL	148.97 ± 5.56	134.17 ± 9.34	0.194
C15:0, μg/mL	217.75 ± 15.89^a^	168.28 ± 15.22^b^	0.037
C16:1n7, μg/mL	262.56 ± 27.15	339.91 ± 25.07	0.051
C16:0, μg/mL	352.73 ± 41.30^a^	176.45 ± 10.59^b^	< 0.01
C17:1n7, μg/mL	6.69 ± 0.97	5.95 ± 0.78	0.561
C17:0, μg/mL	185.33 ± 21.67	217.89 ± 15.73	0.240
C18:3n6, μg/mL	140.62 ± 21.09	144.02 ± 22.00	0.912
C18:3n3, μg/mL	155.86 ± 12.15	169.13 ± 14.26	0.487
C18:2n6c, μg/mL	17.06 ± 2.27	21.84 ± 2.17	0.146
C18:2 (9-CIS, 11-TRANS), μg/mL	209.18 ± 22.78	210.73 ± 21.94	0.961
C18:2 (10-TRANS, 12-CIS), μg/mL	411.54 ± 25.98^b^	557.61 ± 20.71^a^	< 0.01
C18:1n9c, μg/mL	280.23 ± 47.01	262.61 ± 41.80	0.783
C18:1n9t, μg/mL	379.41 ± 48.63	449.88 ± 65.07	0.397
C18:0, μg/mL	331.22 ± 24.91^a^	236.15 ± 25.34^b^	0.015
C20:5n3 (EPA), μg/mL	332.66 ± 30.20	340.06 ± 15.55	0.831
C20:4n6 (ARA), μg/mL	11.11 ± 2.19	12.58 ± 1.47	0.584
C20:3n3, μg/mL	8.97 ± 1.29^b^	18.44 ± 1.02^a^	< 0.01
C20:3n6, μg/mL	92.46 ± 9.34	116.89 ± 11.79	0.122
C20:2, μg/mL	198.61 ± 17.17	229.30 ± 12.14	0.162
C20:1, μg/mL	31.86 ± 5.44^b^	66.81 ± 5.68^a^	< 0.01
C20:0, μg/mL	84.64 ± 7.81	86.01 ± 7.87	0.903
C21:0, μg/mL	358.07 ± 24.92^b^	432.45 ± 24.02^a^	0.045
C22:6n3 (DHA), μg/mL	53.05 ± 5.13	50.09 ± 6.46	0.724
C22:5n3 (DPA), μg/mL	14.08 ± 1.25^b^	25.27 ± 2.10^a^	< 0.01
C22:5n6, μg/mL	32.13 ± 3.04	39.31 ± 3.98	0.169
C22:1n9, μg/mL	25.65 ± 2.98^b^	42.40 ± 3.21^a^	< 0.01
C22:1 T, μg/mL	16.94 ± 1.26	20.55 ± 2.63	0.232
C22:0, μg/mL	165.36 ± 14.03	161.28 ± 14.72	0.843
C23:0, μg/mL	97.28 ± 8.68	100.20 ± 11.42	0.841
C24:1n9, μg/mL	15.54 ± 1.44	19.74 ± 2.41	0.156
C24:0, μg/mL	108.88 ± 9.53	115.27 ± 10.58	0.659
SFA, μg/mL	3199.52 ± 141.26^a^	2795.05 ± 116.15^b^	0.040
UFA, μg/mL	2869.63 ± 99.43^b^	3307.17 ± 106.74^a^	< 0.01
SFA/UFA	1.12 ± 0.05^a^	0.85 ± 0.03^b^	< 0.01
MUFA, μg/mL	1192.31 ± 69.41	1371.89 ± 85.60	0.121
PUFA, μg/mL	1677.32 ± 54.80^b^	1935.28 ± 45.57^a^	< 0.01
CLA^2^, μg/mL	620.72 ± 43.43^b^	768.34 ± 28.42^a^	0.011

SFA, saturated fatty acids; UFA, unsaturated fatty acids; MUFA, monounsaturated fatty acids; PUFA, polyunsaturated fatty acids; CLA, conjugated linoleic acids.

^a,b^Mean values within a row with different superscripts differed (*p* < 0.05).

1 CON, control diet with no CB supplementation; CB, control diet with 20 g/d CB supplementation for per buffalo.

2 CLA included the C18:2 (9-CIS, 11-TRANS) and C18:2 (10-TRANS, 12-CIS) fatty acids.

### 3.6 Blood biochemical indices, hormone levels, oxidative status and immune indices

Table 6 shows that, compared to the CON diet, the CB diet significantly increased (*p* < 0.05) the serum concentration of TP in dairy buffaloes; serum ALB concentration also tended to increase (*p* = 0.072) with CB supplementation. However, no significant differences were observed in other detected blood biochemical parameters between buffaloes fed the CON and CB diets (*p* > 0.05). Similarly, dietary CB failed to influence serum levels of hormones such as INS, T3, and T4 in dairy buffaloes (*p* > 0.05). In addition, compared to the CON group, CB supplementation significantly increased the plasma concentration of CAT (*p* < 0.01); however, plasma SOD activity and T-AOC and MDA levels were not affected by CB supplementation (*p* > 0.05). Regarding serum immune indices, dietary CB supplementation significantly increased serum concentrations of IgM and HSP70, while significantly decreasing serum concentrations of TNF-α, IL-1β, and IL-6 (*p* < 0.05).

**Table 6:** Effects of dietary with *Citrus* bioflavonoids (CB) supplementation on blood biochemical indices, hormone levels, oxidative status, and immune indices of dairy buffaloes during hot weather.

Items	Treatment groups^1^		*p*-value
	CON	CB	
Biochemical indices			
TP, g/L	72.16 ± 1.79^b^	77.76 ± 1.14^a^	0.018
ALB, g/L	37.03 ± 0.93	39.04 ± 0.53	0.072
GLO, g/L	35.12 ± 1.55	37.73 ± 1.45	0.236
A:G	1.09 ± 0.05	1.04 ± 0.05	0.504
ALT, U/L	52.38 ± 2.51	53.90 ± 3.02	0.713
AST, U/L	137.00 ± 5.66	147.50 ± 7.08	0.270
AST:ALT	2.56 ± 0.14	2.82 ± 0.22	0.353
ALP, U/L	173.44 ± 16.75	190.38 ± 23.06	0.555
CK, U/L	168.78 ± 13.21	162.89 ± 10.26	0.729
LDH, U/L	596.88 ± 19.31	602.80 ± 41.27	0.899
UN, mmol/L	6.21 ± 0.28	6.34 ± 0.29	0.750
GLU, mmol/L	2.32 ± 0.22	2.57 ± 0.13	0.839
TC, mmol/L	2.98 ± 0.10	2.93 ± 0.15	0.798
TG, mmol/L	0.23 ± 0.04	0.23 ± 0.03	0.933
Hormone levels			
INS, uIU/mL	7.01 ± 0.70	6.83 ± 074	0.865
T_3_, ng/mL	1.14 ± 0.04	1.18 ± 0.11	0.771
T_4_, ug/dL	4.24 ± 0.28	3.95 ± 0.27	0.471
Oxidative status			
SOD, U/mL	23.01 ± 2.57	26.24 ± 0.62	0.447
T-AOC, nmol/mL	799.40 ± 20.43	807.73 ± 13.10	0.736
CAT, U/mL	1.80 ± 0.21^b^	2.91 ± 0.26^a^	< 0.01
MDA, nmol/mL	1.75 ± 0.21	1.70 ± 0.13	0.810
Immune indices			
IgG, mg/dL	0.088 ± 0.003	0.079 ± 0.005	0.157
IgM, mg/dL	0.50 ± 0.05^b^	0.62 ± 0.03^a^	0.048
IgA, mg/dL	0.044 ± 0.002	0.047 ± 0.003	0.522
SAA, μg/mL	2.63 ± 0.15	2.64 ± 0.11	0.978
HSP70, pg./mL	35.92 ± 1.93^b^	42.42 ± 1.36^a^	0.012
TNF-α, pg./mL	17.69 ± 0.51^a^	14.62 ± 0.69^b^	< 0.01
IFN-γ, pg./mL	347.75 ± 20.65	342.33 ± 14.71	0.831
IL-1β, pg./mL	143.32 ± 4.28^a^	126.60 ± 5.87^b^	0.035
IL-2, pg./mL	56.42 ± 6.31	55.07 ± 3.12	0.851
IL-6, pg./mL	167.62 ± 8.98^a^	140.15 ± 6.15^b^	0.024

TP, total protein; ALB, albumin; GLO, globulin; A:G, albumin-to- globulin ratio; ALT, alanine aminotransferase; AST, aspartate aminotransferase; ALP, alkaline phosphatase; CK, creatine kinase; LDH, lactate dehydrogenase; UN, urea nitrogen; GLU, glucose; TC, total cholesterol; TG, triglyceride; INS, insulin; T3, triiodothyronine; T4, tetraiodothyronine; SOD, superoxide dismutase; T-AOC, total antioxidant capacity; CAT, catalase; MDA, malondialdehyde; IgG, immunoglobulin G; IgM, immunoglobulin M; IgA, immunoglobulin A; SAA, serum amyloid-A; HSP70, heat shock protein 70; TNF-α, tumor necrosis factor-α; IFN-γ, interferon-γ; IL-1β, interleukin-1β; IL-2, interleukin-2; IL-6, interleukin-6.

^a,b^Mean values within a row with different superscripts differed (*p* < 0.05).

1 CON, control diet with no CB supplementation; CB, control diet with 20 g/d CB supplementation for per buffalo.

### 3.7 Fermentation profile in the ruminal contents

Regarding the ruminal fermentation profile, the concentrations of TVFA, acetate, propionate, isobutyrate, butyrate, isovalerate, and valerate, along with the A:*P* value were not significantly altered by CB supplementation (*p* > 0.05; Table 7). Moreover, CB supplemention had no significant effect on ruminal fluid pH or NH_3_-N concentration (*p* > 0.05), whereas it significantly increased (*p* < 0.05) ruminal MCP concentration.

**Table 7:** Effects of dietary with *Citrus* bioflavonoids (CB) supplementation on fermentation profile in the rumen of dairy buffaloes during hot weather.

Items	Treatment groups^1^		*p*-value
	CON	CB	
pH	6.76 ± 0.10	6.89 ± 0.08	0.346
NH_3_-N, mg/dL	8.71 ± 0.44	7.20 ± 1.03	0.233
MCP, mg/dL	12.25 ± 0.33^b^	14.36 ± 0.60^a^	0.015
TVFA, mmol/L	84.32 ± 1.46	78.16 ± 7.36	0.455
Acetate, mmol/L	56.86 ± 2.15	50.96 ± 4.11	0.214
Propionate, mmol/L	14.55 ± 0.51	14.22 ± 1.59	0.852
Isobutyrate, mmol/L	1.21 ± 0.07	1.35 ± 0.17	0.503
Butyrate, mmol/L	8.95 ± 0.44	8.78 ± 1.17	0.894
Isovalerate, mmol/L	1.49 ± 0.09	1.62 ± 0.24	0.634
Valerate, mmol/L	1.25 ± 0.03	1.23 ± 0.18	0.918
A: P	3.95 ± 0.26	3.63 ± 0.14	0.324

NH_3_-N, ammonia-nitrogen; MCP, microbial crude protein; TVFA, total volatile fatty acid; A:P, acetate-to-propionate ratio.

^a,b^Mean values within a row with different superscripts differed (*p* < 0.05).

1 CON, control diet with no CB supplementation; CB, control diet with 20 g/d CB supplementation for per buffalo.

### 3.8 Taxonomic configuration of ruminal bacteria

**Figure 2:**
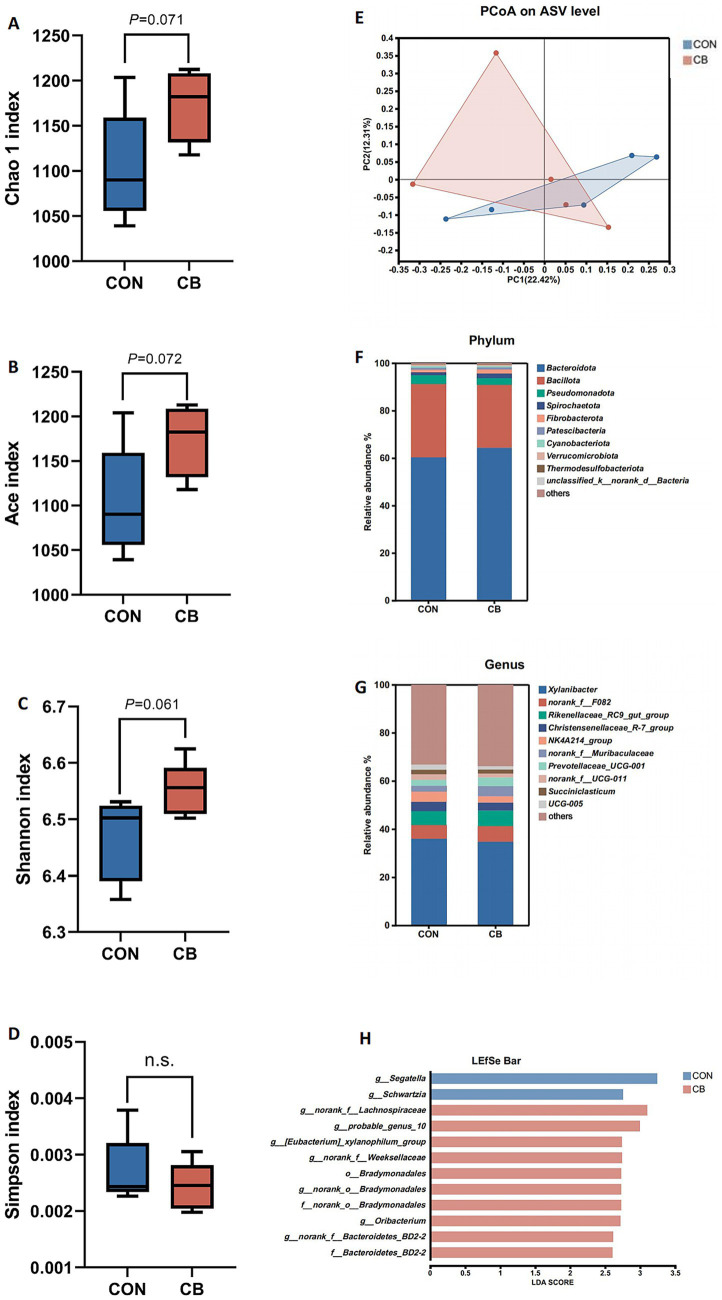
Effects of dietary with *Citrus* bioflavonoids (CB) supplementation on diversity and composition of the rumen bacterial community of dairy buffaloes during hot weather. **(A)** Chao1 index. **(B)** Ace index. **(C)** Shannon index. **(D)** Simpson index. **(E)** Beta diversity based on the principal coordinate analysis (PCoA) using the distance matrices generated from Bray-Curtis analysis. **(F)** Relative abundance of bacteria community at the phylum level. **(G)** Relative abundance of bacteria community at the genus level. **(H)** Linear discriminant analysis effect size approach identifying biomarker genera between two groups. CON, control diet with no CB supplementation; CB, control diet with 20 g/d CB supplementation for per buffalo.

The effect of CB supplementation on ruminal microbes in dairy buffaloes was evaluated using 16S rRNA high-throughput sequencing. As shown in Figures 2A–D, the *α*-diversity indices of CB group, including species richness (Chao 1 and Ace indices), tended to increase (*p* = 0.071 and *p* = 0.072, respectively) compared to the CON group, and bacterial diversity (Shannon index) exhibited a similar trend. No differences were found in the Simpson index between the CON and CB groups (*p* > 0.10). Furthermore, *β*-diversity of rumen bacteria was determined via PCoA, which explained 22.42 and 12.31% of the variance, respectively, however, no distinct separation between the CON and CB groups was observed (Figure 2E).

As illustrated in Figure 2F, *Bacteroidota* (62.35%), *Bacillota* (28.74%), *Pseudomonadota* (3.18%), *Spirochaetota* (1.61%), and *Fibrobacterota* (1.35%) were the dominant bacterial phyla in both groups, with no significant differences in phyla relative abundance between the CON and CB groups (*p* > 0.05, Table 8). At the genus level, the ruminal microbiome was dominated by *Xylanibacter* (35.31%), *norank_f__F082* (6.13%), *Rikenellaceae_RC9_gut_group* (6.09%), *Christensenellaceae_R-7_group* (3.65%), *NK4A214_group* (3.43%), *norank_f__Muribaculaceae* (3.28%), and *Prevotellaceae_UCG-001* (3.08%) (Figure 2G). Compared to the CON group, CB supplementation significantly decreased the relative abundance of *Segatella* in dairy buffaloes (*p* < 0.01; Table 9). Notably, the CB group exhibited an approximately 83.16% increase in the relative abundance of *Fibrobacterota* (phyla level; Table 8) as well as an 82.11% increase in the relative abundance of *Fibrobacter* (genus level; Table 9) compared to the CON group, although these increases did not reach statistical significance.

**Table 8:** Effects of dietary with *Citrus* bioflavonoids (CB) supplementation on bacterial abundance in rumen of dairy buffaloes during hot weather (phylum level, %).

Items	Treatment groups^1^		*p*-value
	CON	CB	
*Bacteroidota*	60.37 ± 4.53	64.32 ± 0.65	0.449
*Bacillota*	30.85 ± 4.10	26.63 ± 0.79	0.382
*Pseudomonadota*	3.64 ± 0.97	2.72 ± 0.42	0.417
*Spirochaetota*	1.34 ± 0.38	1.87 ± 0.40	0.370
*Fibrobacterota*	0.95 ± 0.25	1.74 ± 0.43	0.166
*Patescibacteria*	0.96 ± 0.21	0.94 ± 0.23	0.943
*Cyanobacteriota*	0.66 ± 0.15	0.69 ± 0.27	0.910
*Verrucomicrobiota*	0.56 ± 0.19	0.37 ± 0.07	0.396
*Thermodesulfobacteriota*	0.15 ± 0.04	0.20 ± 0.03	0.356
*unclassified_k__norank_d__Bacteria*	0.14 ± 0.02	0.15 ± 0.02	0.957

1 CON, control diet with no CB supplementation; CB, control diet with 20 g/d CB supplementation for per buffalo.

**Table 9:** Effects of dietary with *Citrus* bioflavonoids (CB) supplementation on bacterial abundance in rumen of dairy buffaloes during hot weather (genus level, %).

Items	Treatment groups^1^		*p*-value
	CON	CB	
*Xylanibacter*	35.88 ± 5.39	34.74 ± 3.01	0.860
*norank_f__F082*	5.78 ± 0.66	6.47 ± 0.85	0.543
*Rikenellaceae_RC9_gut_group*	5.74 ± 0.78	6.44 ± 0.63	0.517
*Christensenellaceae_R-7_group*	3.93 ± 0.84	3.37 ± 0.45	0.579
*NK4A214_group*	4.25 ± 1.06	2.60 ± 0.38	0.193
*norank_f__Muribaculaceae*	2.36 ± 0.36	4.20 ± 2.24	0.505
*Prevotellaceae_UCG-001*	2.52 ± 0.29	3.64 ± 0.75	0.211
*norank_f__UCG-011*	2.32 ± 0.31	1.56 ± 0.21	0.090
*UCG-005*	2.10 ± 0.37	1.42 ± 0.18	0.153
*Succinivibrionaceae_UCG-002*	1.46 ± 0.16	1.38 ± 0.33	0.830
*Succiniclasticum*	1.89 ± 0.15	1.69 ± 0.33	0.600
*Acetobacter*	1.51 ± 0.81	0.69 ± 0.25	0.373
*Ruminococcus*	1.73 ± 0.44	1.11 ± 0.21	0.243
*norank_o__Bacteroidales*	1.24 ± 0.12	1.46 ± 0.14	0.284
*Fibrobacter*	0.95 ± 0.25	1.73 ± 0.44	0.167
*Treponema*	1.18 ± 0.37	1.67 ± 0.39	0.406
*Prevotellaceae_UCG-003*	1.00 ± 0.18	1.32 ± 0.14	0.222
*unclassified_f__Prevotellaceae*	0.93 ± 0.19	1.21 ± 0.16	0.303
*Saccharofermentans*	0.93 ± 0.06	0.91 ± 0.10	0.833
*Segatella*	0.72 ± 0.06^a^	0.36 ± 0.03^b^	< 0.01

^a,b^Mean values within a row with different superscripts differed (*p* < 0.05).

1 CON, control diet with no CB supplementation; CB, control diet with 20 g/d CB supplementation for per buffalo.

Additionally, LEfSe analysis (LDA > 2.5, *p* < 0.05) was used to identify significantly differential bacterial taxa between the CON and CB groups, with 12 biomarkers identified. Increased abundance of members of the dominant *Bacteroidota* phylum was evident in response to CB supplementation, including the family *Bacteroidetes_BD2-2* as well as genera such as *norank_f__Bacteroidetes_BD2-2* and *norank_f__Weeksellaceae*. Besides, the dominant *Bacillota* phylum was also enriched in the CB-supplemented group, including genera such as *norank_f__Lachnospiraceae*, *probable_genus_10, [Eubacterium]_xylanophilum_group*, and *Oribacterium*, all belonging to the family *Lachnospiraceae*. Moreover, the order *Bradymonadales* and its members (including *f__norank_o__Bradymonadales* and *g__norank_o__Bradymonadales*) were enriched in the CB group. In contrast, the CON group exhibited a higher differential abundance of the genera *Segatella* and *Schwartzia* (Figure 2H).

## 4 Discussion

Our laboratory’s previous findings showed that the most accurate model, based on BST and RR, effectively indicates thermal comfort in dairy buffaloes under hot and humid climates, and this model was developed accordingly (13). Using this established thermal comfort index, we confirmed that the dairy buffaloes in the present experiment suffered from relatively severe and persistent heat stress. Phytogenics-derived flavonoids, such as those from mulberry leaves and propolis extract, have emerged as promising modulators for alleviating heat stress responses in ruminants, with accumulating evidence supporting their ability to mitigate physiological perturbations induced by heat stress (4, 14, 15). Heat stress triggers a series of adaptive mechanisms in livestock, including increased RR, a primary thermoregulatory response to dissipate excess body heat (15). As expected, dietary supplementation with CB reduced the RR and BST of dairy buffaloes under hot weather conditions in the present study, and such reductions are indicative of heat stress mitigation (16). Regrettably, however, this study only tested a single CB dose without multi-dose groups, we cannot identify the optimal CB dose for heat-stressed dairy buffaloes or clarify whether CB exert dose-dependent effects on heat stress alleviation. In terms of nutrient digestibility, our study demonstrated that CB supplementation improved the apparent digestibility of ADF without affecting DMI of dairy buffaloes, which is in accordance with others studies, who reported that feeding *Citrus* extracts did not alter DMI of dairy cows (11, 17). The absence of DMI changes in our study suggests that the improved ADF digestibility and subsequent benefits to buffaloes’ lactation performance are not driven by increased feed consumption but rather by enhanced nutrient utilization efficiency. Given that ADF digestion relies on the synergistic action of enzymes and rumen microorganisms, the improved ADF digestibility in our study may reflect CB-induced modifications in cellulase activity or rumen microbial communities.

Heat stress poses a major challenge to dairy animals, leading to deteriorated lactation performance and altered metabolic processes. This is typically accompanied by reductions in milk protein, milk fat, lactose, SNF, and TS contents, along with increased MUN levels, collectively resulting in diminished milk quality (18–20). Accumulating evidence has demonstrated that feeding *Citrus* extract exerts positive effects on milk production and composition in dairy cows (11, 21). Consistent with these findings, our study observed higher milk yield, 4%FCM, milk protein, milk lactose, and SNF in the CB-supplemented group. These results indicate that dietary CB supplementation significantly improves the milk quality of dairy buffaloes under high-temperature conditions. However, in contrast to our findings, Ying et al. (17) reported no significant effects of *Citrus* extract supplementation on milk yield or composition. Notably, the supplementation level employed in their research (4 g/d) was substantially lower than that used in ours, and this dosage discrepancy may well explain the differing outcomes regarding lactation performance. Currently, studies investigating the effects of flavonoid-rich plant extracts on the milk fatty acids profile of ruminants remain limited. Dietary phytogenic compounds can optimize milk fatty acid composition by regulating microbial biohydrogenation in the rumen (22). In the present study, CB treatment resulted in an approximately 15% increase in UFA and a 24% increase in CLA, coupled with reduced levels of major SFA, including myristic acid (C14:0) and palmitic acid (C16:0). These changes are desirable from a human health perspective, with potential benefits particularly in terms of cardiovascular protection and metabolic regulation. These findings can be reasonably explained by the ability of flavonoids to selectively modulate specific rumen microorganisms, thereby reducing the biohydrogenation of ingested fatty acids and increasing the proportion of UFA (23). For instance, condensed tannins, a class of flavonoid-based polymers, have been shown to partially inhibit the final step of C18:3 biohydrogenation in the Rumen Simulation Technique system (24). The observed decrease in stearic acid (C18:0) alongside increased CLA content in our study is consistent with earlier research, which reported similar results in dairy ewes supplemented with tannins (25). It is generally hypothesized that tannins enhance the activity of stearoyl-CoA desaturase (SCD), an enzyme that mediates the conversion of stearic acid to oleic acid and vaccenic acid to CLA. This implies that flavonoids can increase milk UFA levels not only by regulating rumen biohydrogenation but also by enhancing SCD activity (25, 26). However, due to the lack of data on fatty acid content in rumen microbial biomass in the current study, we were unable to establish a direct link between microbial abundance and milk fatty acid profiles. This aspect warrants further investigation in future research. From the perspective of application prospects, CB show great potential as a green feed additive in future livestock farming, its ability to alleviate heat stress and improve lactation performance makes it particularly suitable for high-yielding lactating ruminants in tropical/subtropical regions, where heat stress often limits production.

The CB supply did not interfere with the most of biochemical parameters, except for TP and ALB, whose concentrations were higher in the CB group compared to the CON group. Consistent with our findings, previous studies have also reported that flavonoid-rich extracts, such as those from mulberry leaves (13) or propolis (27), exert little to no impact on the blood concentrations of common biochemical parameters in heat-stressed dairy cows or buffaloes. However, in contrast to the current study, which observed no significant effects on metabolic hormones, some prior research has documented flavonoids-induced changes in hormonal parameters. For instance, mulberry leaf flavonoids supplementation in buffaloes was shown to significantly alter serum insulin, T3, and T4 levels (13). This discrepancy suggests that the effects of flavonoids on hormonal parameters may be influenced by factors such as flavonoid type/dosage or the supplementation duration. Under normal environmental conditions, the oxidant-antioxidant system maintains a delicate balance to sustain bodily homeostasis. However, exposure to elevated ambient temperature disrupts this equilibrium, triggering oxidative stress. Heat stress induces an excessive accumulation of ROS in animals, leading to severe oxidative stress that subsequently accelerates lipid peroxidation and impairs immune responses (28, 29). Therefore, maintaining oxidant-antioxidant balance is critical to mitigating the adverse effects of oxidative stress. In dairy animals, particularly dairy buffaloes, the impact of oxidative stress is more pronounced, as milk production, a metabolically active process, generates additional heat, exacerbating the stress burden. Under oxidative stress conditions, the body’s defense system fails to timely scavenge excessive free radicals, primarily due to reduced activities of antioxidant enzymes such as CAT, SOD, and GSH-Px during chronic heat stress (30, 31). Consequently, the activity of these antioxidant enzymes in ruminants is widely used as physiological indicator to assess the severity of oxidative and heat stress (30). As a comprehensive marker of the antioxidant system, plasma T-AOC reflects the cumulative effect of all antioxidants in the body, with higher levels indicating stronger antioxidant capacity (32). Conversely, MDA, a byproduct of lipid peroxidation, serves as a key indicator of oxidative damage, where lower plasma MDA concentrations signify reduced oxidative stress (33). Previous studies have confirmed that numerous traditional Chinese medicinal materials can enhance the antioxidant capacity of dairy ruminants under heat stress. For instance, dietary supplementing of dairy cows with *Radix bupleuri* or *Lonicera japonica* has been shown to increase plasma SOD activity and T-AOC levels, while simultaneously reducing MDA concentrations (34, 35). Similarly, a study on multiparous Murrah buffaloes demonstrated that mulberry flavonoids inhibited MDA in a dose-dependent manner, exerting a positive effect on alleviating oxidative stress (12). Consistent with these findings, the present study observed a remarkable increase (up to 61.67%) in plasma CAT activity following CB supplementation, indicating CB’s potential to alleviate heat-induced oxidative stress. This effect is likely attributed to the ability of flavonoids to act as reducing agents and hydrogen donors, which neutralize ROS and scavenge hydrogen peroxide and superoxide ions (36). HSP70, known as a cellular “thermometer” for mediating heat stress, is pivotal for thermotolerance and plays critical roles in cellular heat resistance, immune modulation, and heat stress responses (37). In the present study, under heat stress conditions, CB supplementation significantly increased serum HSP70 content in buffaloes compared to the CON group. Earlier studies using a mouse model reported that quercetin can effectively upregulate HSP70 expression by mediating the ERK/PPARγ signaling pathways (38). Biomarkers of inflammatory status provide direct insights into homeostasis and health in dairy ruminants (39). Indeed, the elevation and release of proinflammatory cytokines are hallmarks of oxidative stress, associated with mitochondrial dysfunction and increased free radical production (40, 41). Notably, the observed increase in serum IgM levels, alongside reduced pro-inflammatory cytokines (e.g., TNF-α, IL-1β, and IL-6) suggests that CB supplementation can enhance immune function while alleviating inflammatory response in buffaloes during hot weather. This aligns with recent studies on *Citrus* extract (containing hesperidin and naringin) in dairy cows, which demonstrated reduced systemic inflammation, evidenced by lower serum endotoxin, IL-1β and TNF-α levels, indicating the potential anti-inflammatory effects of *Citrus* extract and improved immune-metabolic status in dairy cows (10, 11).

It is widely accepted that VFA act as the primary carbon source for ruminal microbes and provide 60% ~ 80% of the digestible energy required by ruminants (42). Furthermore, the concentration and composition of VFA are key indicators of rumen fermentation status and microbial community structure. A growing body of evidence has shown that dietary supplementation with flavonoid-rich extracts can alter the concentration or composition of rumen VFA (43–45). However, our observations revealed that CB supplementation had no significant effect on either total or individual VFA concentrations in buffaloes under hot climatic conditions. These findings add to the accumulating literature indicating that plant-derived flavonoids exert variable effects on rumen fermentation, with such inconsistencies likely stemming from differences in flavonoid sources, dosage, animal species, and experimental conditions. Encouragingly, our results demonstrated that CB supplementation increased ruminal MCP concentration in heat-stressed buffaloes, suggesting that the effects of CB are more specific to microbial protein synthesis rather than broad fermentation changes. To our knowledge, flavonoid-rich extracts have been associated with elevated MCP concentrations, potentially by creating a favorable microenvironment for ruminal microbiota involved in protein metabolism or serving as an energy substrate for microbial growth, thereby enhancing nitrogen utilization efficiency (46, 47). This is supported by the present study, where CB treatment significantly increased both MCP and milk protein contents in the experimental buffaloes.

A stable and diverse rumen microbial community is fundamental to the health and productivity of ruminants, as it provides functional redundancy and enhances resilience against dietary or environmental perturbations (48). In our study, three key microbial *α*-diversity indices, Chao 1, ACE, and Shannon, all exhibited an upward trend. This observation is particularly vital, as it implies that the rumen microbial community in the CB group was not only richer in species but also more evenly distributed in species abundance. A more evenly distributed microbial community is generally regarded as more stable, primarily because it is less prone to collapse if one or two dominant species are adversely affected. Notably, this CB-induced enhancement of microbial diversity contrasts sharply with the effects of some potent antimicrobial feed additives (e.g., monensin). Monensin is known to occasionally reduce overall rumen microbial diversity by specifically targeting gram-positive bacteria (49). The effect of CB aligns more closely with certain other flavonoids that have been shown to modulate the rumen microbiome without compromising its overall diversity, thereby fostering a healthier and more robust fermentation system (50).

Beyond improving diversity, the >80% increase in the relative abundance of the phylum *Fibrobacterota*, along with its primary genus, *Fibrobacter*, in the CB group is of paramount importance. *Fibrobacter* species are recognized as keystone cellulolytic bacteria in the rumen, equipped with highly efficient enzyme systems that enable the degradation of crystalline cellulose, one of the most recalcitrant components of plant cell walls (51). The enrichment of *Fibrobacter* strongly suggests that CB enhance the host’s capacity to digest low-quality forage, which in turn facilitates greater energy release in the form of VFA. This effect is particularly valuable for high-producing lactating ruminants, where energy demand is a primary limiting factor for milk production. Furthermore, LEfSe analysis further validated the pro-fibrolytic role of CB by identifying the phylum *Bacillota*, and specifically the family *Lachnospiraceae*, as significantly enriched in the CB group. Within *Lachnospiraceae*, the [*Eubacterium*]*_xylanophilum_group*, is specialized in degrading xylan, a major component of hemicellulose. Its enrichment complements the cellulolytic activity of *Fibrobacter*, ensuring the comprehensive breakdown of complex plant fibers. Similarly, the family *Bacteroidetes_BD2-2* and the genus *norank_f__Weeksellaceae*, both members of the phylum *Bacteroidota*, were also enriched in the CB group. *Bacteroidota* is well-documented for its extensive repertoire of carbohydrate-active enzymes, which enable the degradation of a broad range of polysaccharides (52). Collectively, the enrichment of these taxa further reinforces the hypothesis that CB enhance the overall glycan-degrading capacity of the rumen microbiome, a mechanism that may partially explain the improved ADF digestibility observed in CB-supplemented dairy buffaloes. In addition to promoting fibrolytic bacteria, our data show that CB selectively suppress specific microbial groups, particularly those involved in protein and amino acid metabolism. LEfSe analysis revealed that the genera *Segatella* and *Schwartzia* were significantly more abundant in the CON group. By suppressing *Segatella*, CB may help preserve dietary protein, thereby increasing the flow of MCP and bypass protein to the small intestine. This, in turn, supports more efficient nitrogen absorption and utilization for milk protein synthesis. This mechanism aligns with findings from studies on other flavonoids, such as biochanin A (a key isoflavone in the pasture legume red clover), which have been shown to inhibit hyper-ammonia-producing bacteria and improve nitrogen utilization efficiency in ruminants (53). Overall, dairy CB the supplementation orchestrates a beneficial and comprehensive shift in the rumen microbial ecosystem of dairy buffaloes. To further elucidate CB’s mechanisms, future research should integrate multi-omics approaches (e.g., metagenomics, metabolomics) to validate microbial functional pathways and their links to host metabolic changes.

## 5 Conclusion

In this study, we demonstrated that CB exert a distinct beneficial effect on the lactation performance of dairy buffaloes during hot weather, primarily by enhancing their antioxidant capacity and regulating immune function. Meanwhile, dietary CB supplementation was associated with specific shifts in the ruminal microbial communities of dairy buffaloes, namely, the enrichment of fiber-degrading taxa and the suppression of the genus *Segatella*. This microbial shift is likely linked to the observed improvements in ADF digestibility and ruminal MCP concentration. In summary, CB exhibit considerable potential as a natural feed supplement for dairy buffaloes, as it can alleviate heat stress, thereby optimizing ruminal function and further improving lactation performance. Despite these prospects, the large-scale application of CB in livestock farming still faces practical challenges that require addressing, particularly in terms of extraction efficiency, stability during feed processing, and production cost control.

## Data Availability

The datasets presented in this study can be found in online repositories. The names of the repository/repositories and accession number(s) can be found in the article/supplementary material.

## Glossary

ROS: Reactive oxygen species
TMR: Total mixed ration
AT: Air temperature
RH: Relative humidity
RT: Rectal temperature
BST: Body surface temperature
RR: Respiratory rate
ATTD: Apparent total tract digestibility
DM: Dry matter
CP: Crude protein
EE: Ether extract
ADF: Acid detergent fiber
NDF: Neutral detergent fiber
MUN: Milk urea nitrogen
TS: Total solids
SNF: Solids-not-fat
FFA: Free fatty acids
TP: Total protein
ALB: Albumin
ALT: Alanine transaminase
AST: Aspartate aminotransferase
ALP: Alkaline phosphatase
CK: Creatine kinase
LDH: Lactate dehydrogenase
UN: Urea nitrogen
GLU: Glucose
TC: Total cholesterol
TG: Triglyceride
IgG: Immunoglobulin G
IgM: Immunoglobulin M
IgA: Immunoglobulin A
INS: Insulin
T3: Triiodothyronine
T4: Tetraiodothyronine
SOD: Superoxide dismutase
CAT: Catalase
T-AOC: Total antioxidant capacity
MDA: Malondialdehyde
SAA: Serum amyloid A
HSP70: Heat shock protein 70
TNF-α: Tumor necrosis factor-α
IFN-γ: Interferon-γ
IL-1β: Interleukin-1beta
IL-2: Interleukin-2
IL-6: Interleukin-6
VFA: Volatile fatty acids
NH_3_-N: Ammonia nitrogen
MCP: Microbial crude protein
ASV: Amplicon sequencing variants
SFA: Saturated fatty acids
PUFA: Polyunsaturated fatty acids
UFA: Unsaturated fatty acid
MUFA: Monounsaturated fatty acids
SCD: Stearoyl-CoA desaturase
